# Combination of serum 5-S-cysteinyldopa, melanoma inhibitory activity and IL-8 improves the diagnostic accuracy of malignant melanoma compared with individual markers

**DOI:** 10.1097/MD.0000000000030471

**Published:** 2022-09-02

**Authors:** Yuki Katoh, Hiroyuki Hara, Tomonori Harada, Shuichi Hirai

**Affiliations:** a Division of Anatomical Science, Department of Functional Morphology, Nihon University School of Medicine, Itabashi-ku, TokyoJapan; b Department of Obstetrics and Gynecology, Keio University School of Medicine, Shinjuku-ku, TokyoJapan.

**Keywords:** 5-S-cysteinyldopa, interleukin-8, malignant melanoma, melanoma inhibitory activity, serum biomarker combination

## Abstract

Early diagnosis of malignant melanoma is critical for effective treatment and reduced patient mortality. However, current clinical and histological variables show limited accuracy in diagnosis. Serum or urine level of 5-S-cysteinyldopa (5-S-CD) is a commonly used melanoma biomarker in Japan owing to its increased sensitivity compared with other melanoma markers. However, its use as a diagnostic marker has shown some limitations. Therefore, here we examined the combination of 5-S-CD with melanoma inhibitory activity, which showed sensitivity in detecting melanoma comparable with that of 5-S-CD, and interleukin-8, a cytokine linked with melanoma progression, in a cohort of Japanese patients with melanoma. Our results revealed that the triple combination of 5-S-CD, melanoma inhibitory activity, and interleukin-8 showed high diagnostic accuracy in detecting melanoma compared with each of the individual factors. Importantly, the triple marker showed specificity and utility in detecting early-stage melanoma. Our results suggest the utility of the triple marker as a diagnostic biomarker for melanoma patients.

## 1. Introduction

Malignant melanoma is an invasive skin cancer that originates from melanocytes. Its incidence continues to increase worldwide, and it is estimated that by 2040, melanoma will overtake colorectal and lung cancer to become the second most common cancer in the United States.^[1]^ While melanoma that is diagnosed at a late stage has a strong propensity for metastasis, melanoma that is diagnosed and treated early poses a low mortality risk. However, classical clinical and histological variables such as Breslow thickness, presence of ulcers, and lymph node status do not show sufficient accuracy for early diagnosis. Therefore, new biomarkers for melanoma with high accuracy for early diagnosis and prediction of disease progression are required.

5-S-Cysteinyldopa (5-S-CD), the precursor of pheomelanin, is a molecule produced by melanocytes and melanoma cells, which is detected in the urine and serum of melanoma patients.^[2]^ The serum or urine level of 5-S-CD is the most widely used biomarker for melanoma in Japan because it is significantly elevated in the serum of melanoma patients and detectable earlier than physical examinations, laboratory tests, and imaging techniques, and it reflects the progression of melanoma.^[3–6]^ A recent study reported that serum 5-S-CD levels are more sensitive than lactate dehydrogenase, another melanoma marker, in detecting advanced stage (stage III/IV) melanoma, and serum 5-S-CD levels at the time of initial diagnosis correlate with poor prognosis.^[7]^ However, some studies showed that ultraviolet radiation,^[8,9]^ hemodialysis,^[10]^ dihydroxyphenylalanine,^[11]^ mushrooms, and Agaricus intake affect the amount of 5-S-CD in urine and serum, and other reports found that serum 5-S-CD levels are often not elevated in patients with achromatous malignant melanoma^[3,9,12]^ and stage I/II melanoma.^[13]^ These observations limit the use of 5-S-CD as a diagnostic marker for melanoma.

Melanoma inhibitory activity (MIA) is a molecule produced by chondrocytes and melanoma cells and is involved in cell proliferation and cell adhesion.^[14]^ MIA has shown sensitivity for melanoma, comparable with that of 5-S-CD, and can function as a marker for the early detection of postoperative recurrence and metastasis in melanoma patients.^[15–17]^ However, some studies have revealed abnormally high levels of MIA in cancer types other than melanoma and other diseases, such as Von Recklinghausen disease, suggesting limitations for the use of MIA alone in melanoma.^[18]^

Recent reports have shown that the expression of chemokines such as C-C motif chemokine ligand 4, C-C motif chemokine ligand 5, C-X-C motif chemokine ligand 9, and C-X-C motif chemokine ligand 10, which are associated with lymphocyte infiltration, and the expression of cytokines such as interleukin (IL)-6, IL-8, and IL-10 in plasma are altered.^[19,20]^ IL-8 in particular has received much attention because it is a mitogenic and angiogenic factor in melanoma progression and plays an important role in stage progression and metastasis.^[21–23]^ However, to our knowledge, no study has examined the utility of IL-8 as an early diagnostic marker for melanoma.

In this study, we focused on 5-S-CD, MIA, and IL-8 and evaluated their ability as serum factors for the early diagnosis of melanoma patients. Our results revealed that the combination of the 3 factors markedly improved the diagnostic accuracy and enabled early diagnosis compared with the individual factors alone.

## 2. Methods

### 2.1 Patients and blood samples

Melanoma patients who had been treated for primary or recurrent melanoma at the Department of Dermatology of Nihon University Itabashi Hospital during the study period and were able to obtain consent were enrolled in the study. The diagnosis of malignant melanoma was verified by histological examination, and metastases were verified by histology or other imaging procedures (chest X-ray, abdominal ultrasound, magnetic resonance imaging, computerized tomography, or positron emission tomography). The tumor stage was defined following the pTNM classification of the American Joint Committee on Cancer (AJCC). The follow-up time ranged from 4 to 28 months. Blood samples were collected from 64 malignant melanoma patients and 10 healthy volunteers as a control group. Blood samples were centrifuged at 4000*g* for 10 minutes. Serum samples were aliquoted and stored at –70°C until evaluation. All patients provided informed consent for the use of the clinical materials for research purposes. This study was approved by the Institutional Review Board.

### 2.2 Measurement of serum 5-S-CD levels

Determination of 5-S-CD was performed using high-performance liquid chromatography with electrochemical detection (SRL, Inc., Tokyo, Japan). The upper limit of the normal reference range of serum 5-S-CD was 8 nmol/L, and values exceeding this were defined as abnormal.

### 2.3 Measurement of serum MIA levels

MIA was measured using a 1-step enzyme-linked immunosorbent assay (ELISA) (Roche, Mannheim, Germany) with 2 labeled monoclonal antibodies directed against 14-mer peptide sequences at the N-terminal (MAB 1A12) and C-terminal (MAB 2F7). The assay was performed in accordance with the manufacturer’s instructions. All serum samples and standards were measured in duplicate. A value of 9.6 ng/mL or above was considered an abnormal value.

### 2.4 Measurement of serum IL-8 levels

ELISA was carried out using a human IL-8 ELISA kit (Biosource, Inc., Camarillo, CA) in accordance with the manufacturer’s instructions. A value of 7.2 pg/mL or above was considered an abnormal value.

### 2.5 Statistical analysis

All statistical analyses were performed using GraphPad Prism 9 software. Comparisons between 2 groups were assessed using 2-tailed Student *t* tests. One-way analysis of variance test followed by Bonferroni multiple comparisons tests for multiple comparisons were conducted to compare the differences across independent groups. Receiver operating characteristics (ROC) analyses were used to assess diagnostic performance. The area under the ROC curve was determined as the proportion of area of the entire graph that was beneath the curve. An area under the ROC curve of 0.5 to 0.7 was considered as low accuracy, 0.7 to 0.9 as moderate accuracy and 0.9 to 1.0 as high accuracy. ROC curves were used to display the relationship between sensitivity (true-positive rate, y-axis) and 1-specificity (false-positive rate, x-axis). Data are presented as means ± standard error. *P* < .05 was considered statistically significant.

## 3. Results

### 3.1 *Utility of* 5-S-CD, *IL-8 and MIA alone as melanoma serum biomarkers*

**Figure 1.**
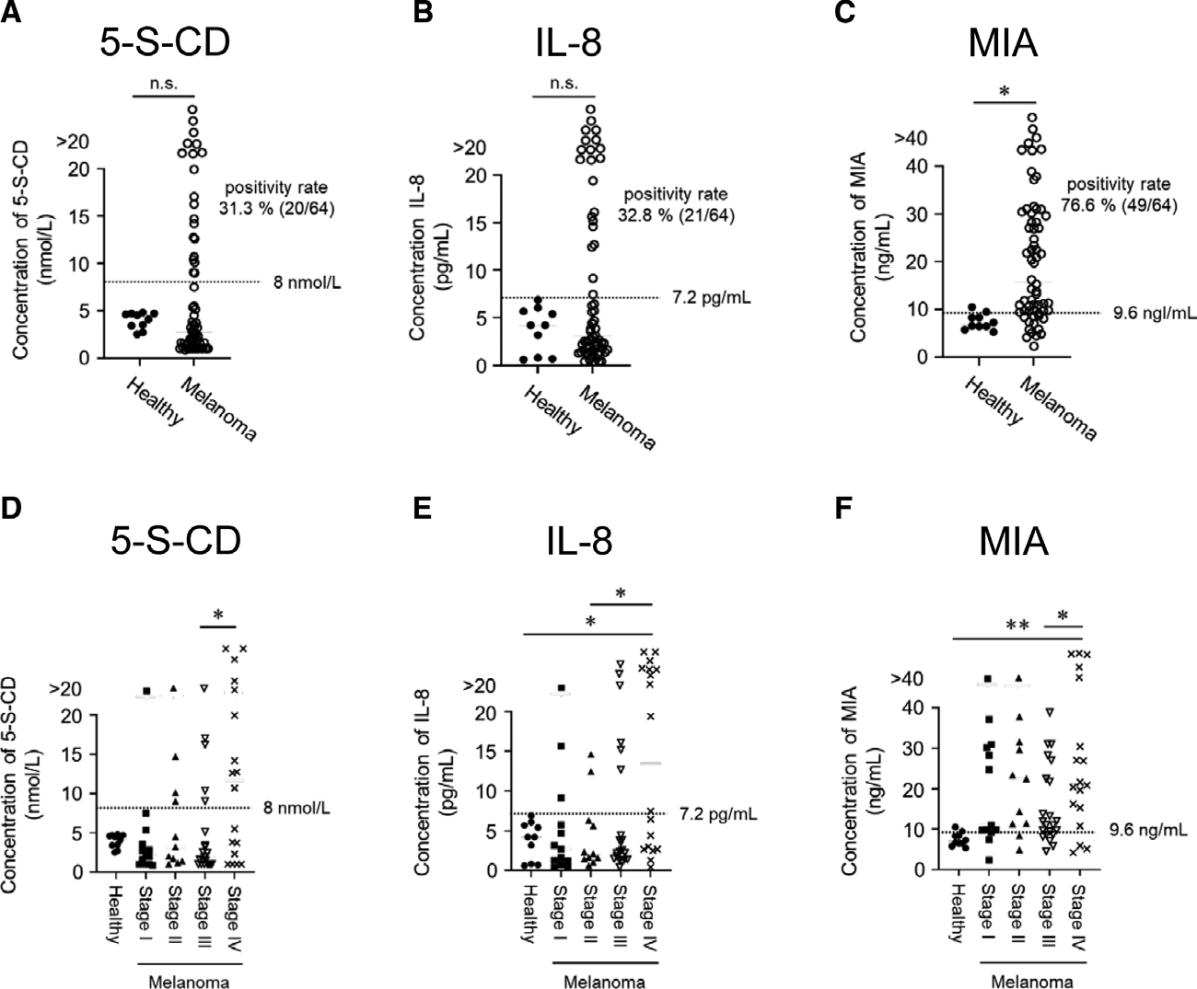
Serum 5-S-CD, IL-8, and MIA levels are higher in melanoma patients than in healthy controls. (A–C) Comparison of serum levels of 5-S-CD (A), IL-8 (B), and MIA (C) in healthy donors (n = 10) with those in patients with stage I–IV melanoma (n = 64). (D–F) Comparison of serum levels of 5-S-CD (D), IL-8 (E), and MIA (F) in healthy donors (n = 10) with those in patients with stage I (n = 13), stage II (n = 11), stage III (n = 22), and stage IV (n = 18) melanoma. The dotted lines indicate the threshold values for each factor. Statistical analysis was performed using the 1-way ANOVA test followed by Bonferroni multiple comparison test. *P < .05, **P < .01. n.s., not significant. ANOVA = analysis of variance, 5-S-CD = 5-S-cysteinyldopa, IL-8 = interleukin 8, MIA = melanoma inhibitory activity, ROC = receiver operating characteristics.

We first measured the levels of 5-S-CD, MIA, and IL-8 in the sera of melanoma patients to evaluate the effectiveness of each individual factor as a serum biomarker. The positivity rate of 5-S-CD (threshold: 8.0 nmol/L) was 31.3% (Fig. 1A). IL-8 (threshold: 7.2 pg/mL) showed a positive rate of 32.8%, which was similar to that of 5-S-CD (Fig. 1B). MIA (threshold: 9.6 ng/mL) had a positive rate of 76.6% (Fig. 1C) and distinguished melanoma patients with high accuracy.

**Figure 2.**
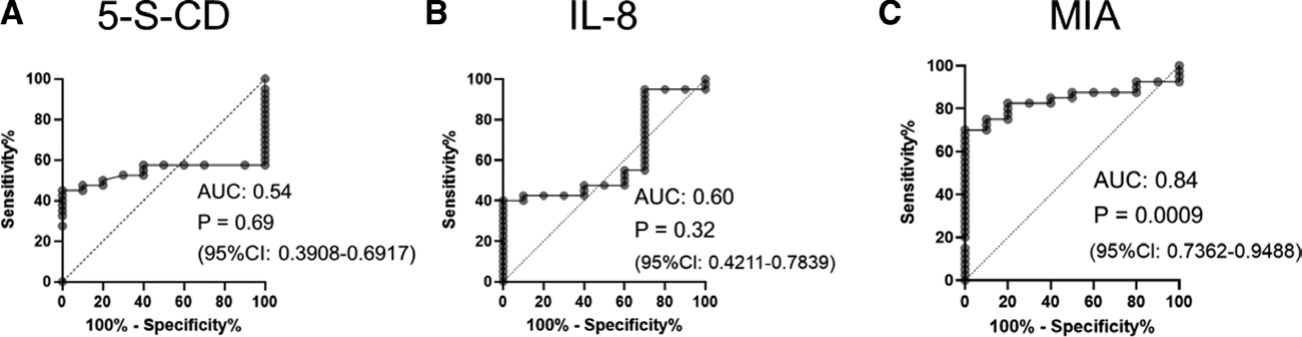
MIA discriminates melanoma patients with high accuracy. ROC curves of serum 5-S-CD, IL-8, and MIA levels. (A) 5-S-CD cutoff point, (B) IL-8 cutoff point, and (C) MIA cutoff point in patients with melanoma. AUC = area under the ROC curve, CI = confidence interval, 5-S-CD = 5-S-cysteinyldopa, IL-8 = interleukin 8, MIA = melanoma inhibitory activity, ROC = receiver operating characteristics.

In the evaluation by stage, as in previous reports,^[3]^ the positive rates of 5-S-CD and IL-8 were low in stage I and II samples, and the positive rate tended to increase as the stage progressed (Fig. 1D, E). Notably, MIA showed a high positivity rate in stage I/II samples (Fig. 1F), and ROC analysis confirmed its usefulness as a diagnostic biomarker (Fig. 2A–C). Detailed data on the ability of each factor to serve as a biomarker are summarized in Table 1.

**Table 1:** Ability of 5-S-CD, IL-8, and MIA as diagnostic biomarkers in melanoma patients.

	5-S-CD				
	Threshold: 8.0 nmol/L				
	Stage I	Stage II	Stage III	Stage IV	Total
Sensitivity (%)	7.7	36.4	22.7	55.6	31.3
Specificity (%)	100	100	100	100	100
False-negative rate (%)	92.3	63.6	77.3	44.4	68.8
False-positive rate (%)	0	0	0	0	0
Positive predictive value (%)	100	100	100	100	100
Negative predictive value (%)	45.5	58.8	37.0	55.6	18.5
Prevalence (%)	56.5	52.4	68.8	64.3	86.5
	IL-8				
	Threshold: 7.2 pg/mL				
	Stage I	Stage II	Stage III	Stage IV	Total
Sensitivity (%)	23.1	18.2	27.3	55.6	32.8
Specificity (%)	100	100	100	100	100
False-negative rate (%)	76.9	81.8	72.7	44.4	67.2
False-positive rate (%)	0	0	0	0	0
Positive predictive value (%)	100	100	100	100	100
Negative predictive value (%)	50.0	52.6	38.5	55.6	18.9
Prevalence (%)	56.5	52.4	68.8	64.3	86.5
	MIA				
	Threshold: 9.6 ng/mL				
	Stage I	Stage II	Stage III	Stage IV	Total
Sensitivity (%)	76.9	81.8	68.2	83.3	76.6
Specificity (%)	90	90	90	90	90
False-negative rate (%)	23.1	18.2	31.8	16.7	23.4
False-positive rate (%)	10	10	10	10	10
Positive predictive value (%)	91	90	94	94	98
Negative predictive value (%)	75.0	81.8	56.3	75.0	37.5
Prevalence (%)	56.5	52.4	68.8	64.3	86.5

5-S-CD = 5-S-cysteinyldopa, IL-8 = interleukin 8, MIA = melanoma inhibitory activity.

### 3.2 *Combination of* 5-S-CD, *IL-8, and MIA as a potential melanoma serum biomarker*

**Figure 3.**
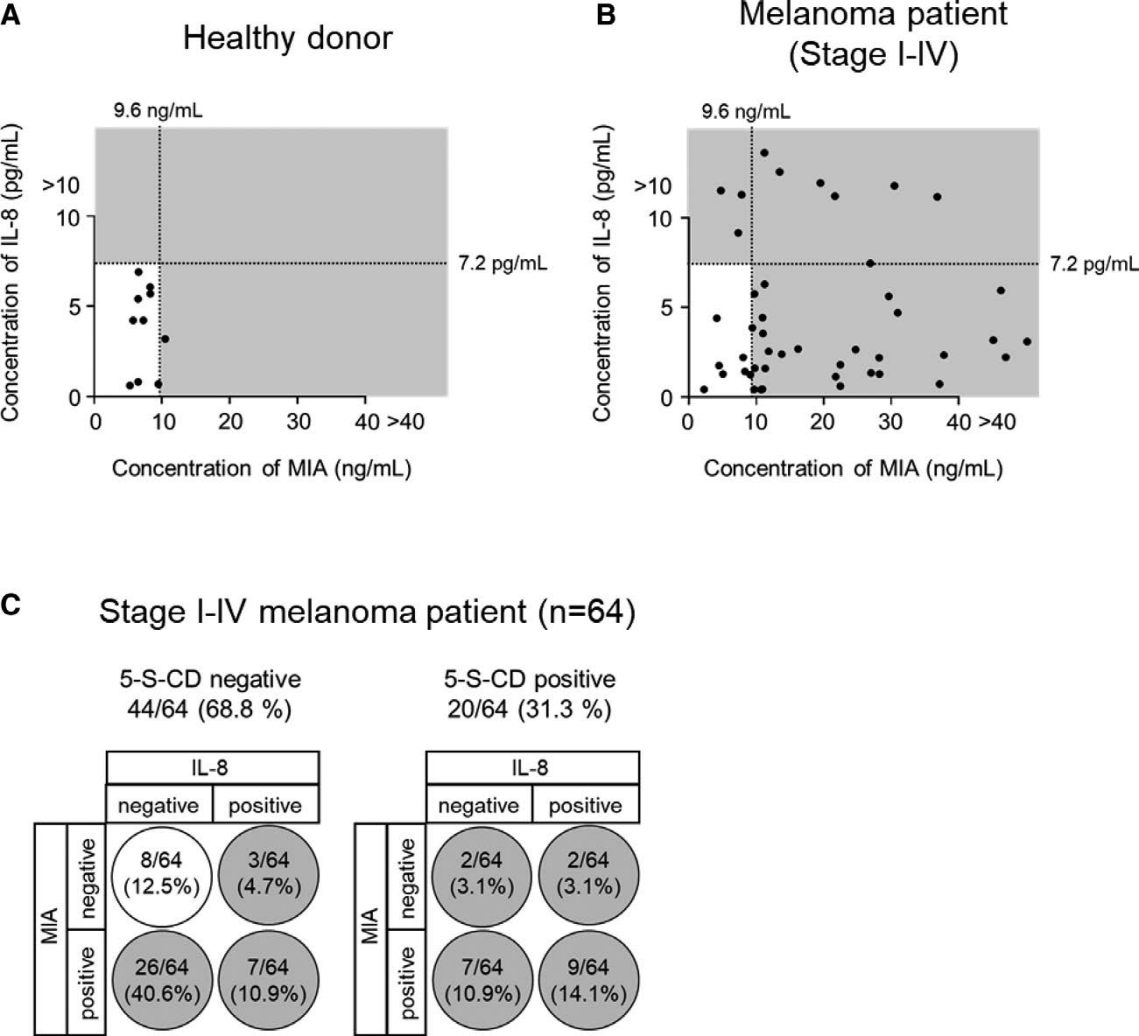
The combination of 5-S-CD, IL-8, and MIA in serum accurately diagnoses stage I–IV melanoma patients. (A, B) Healthy donors (A) and stage I–IV melanoma patients (B) with lower-than-threshold 5-S-CD serum levels were plotted on the basis of IL-8 (y-axis) and MIA (x-axis) levels. Gray areas indicate abnormal levels of IL-8 and/or MIA. (C) Summary of positivity rates by the combination of 5-S-CD, IL-8, and MIA (triple marker) in stage I–IV melanoma patients (n = 64). White circles indicate undetected (false negative) by triple markers, and gray circles indicate detected (positive) by triple markers. 5-S-CD = 5-S-cysteinyldopa, IL-8 = interleukin 8, MIA = melanoma inhibitory activity.

As previously reported,^[4]^ although 5-S-CD has some ability as a serum biomarker for melanoma, 68.8% (44/64) of melanoma cases were undetected by 5-S-CD (Fig. 1A, D). These results indicate the potential for improvement of 5-S-CD as a biomarker, especially in the detection of stage I/II melanoma. Therefore, we investigated whether the combination of the 3 factors, 5-S-CD, IL-8, and MIA, would improve the detection rate. In the 44 cases that were negative for 5-S-CD alone (Fig. 1A, D), the combination of 5-S-CD with IL-8 or MIA led to the detection of 10 or 23 additional cases as positive, respectively. Furthermore, when the combination 5-S-CD, IL-8, and MIA was applied (triple marker), 36 additional cases were positive. In healthy donors, 90% (9/10) were negative and the false-positive rate was low even when the triple marker was used (Fig. 3A, B). These results indicate that the combination of the 3 factors showed improved accuracy compared with each biomarker alone (Fig. 3C).

**Figure 4.**
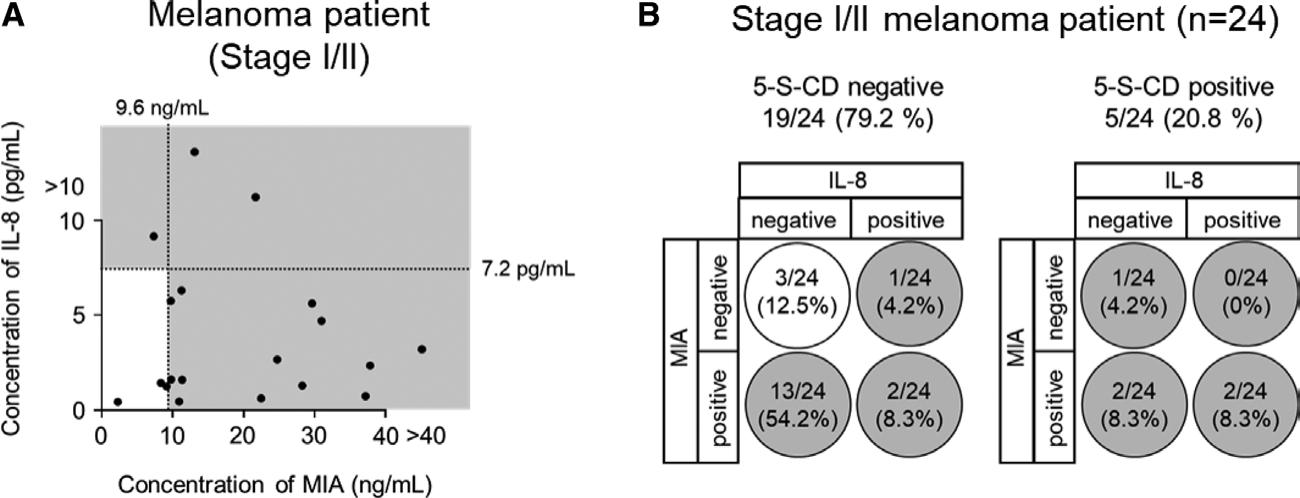
The combination of 5-S-CD, IL-8, and MIA levels in serum is effective in the early diagnosis of melanoma. (A) Stage I/II melanoma patients with lower-than-threshold 5-S-CD were plotted on the basis of IL-8 (y-axis) and MIA (x-axis) levels. Gray areas indicate abnormal levels of IL-8 and/or MIA. (B) Summary of positivity rates by the combination of 5-S-CD, IL-8, and MIA (triple marker) in stage I/II melanoma patients (n = 24). White circles indicate undetected (false negative) by triple markers, and gray circles indicate detected (positive) by triple markers. 5-S-CD = 5-S-cysteinyldopa, IL-8 = interleukin 8, MIA = melanoma inhibitory activity.

We then evaluated the usefulness of the triple marker for patients with stage I/II disease, for whom the positivity rate was low with the use of the single factor. Among the 19 cases that were negative for 5-S-CD alone (Fig. 1D), 3 new cases were detected as positive when analyzed with 5-S-CD and IL-8, and 13 new cases were detected as positive when analyzed with 5-S-CD and MIA. Furthermore, when the triple marker was applied, 16 additional cases were detected as positive (Fig. 4A). Detailed data on the ability of the triple marker to serve as a biomarker are summarized in Table 2. These results suggest that the triple marker may be useful as an early diagnostic marker for melanoma (Fig. 4B).

**Table 2:** Ability of triple marker in stage I (n = 13), stage II (n = 11), stage III (n = 22), and stage IV (n = 18) melanoma patients.

	Triple marker				
	Stage I	Stage II	Stage III	Stage IV	Total
Sensitivity (%)	84.6	90.9	86.4	88.9	87.5
Specificity (%)	90.0	90.0	90.0	90.0	90.0
False-negative rate (%)	15.4	9.1	13.6	11.1	12.5
False-positive rate (%)	10.0	10.0	10.0	10.0	10.0
Positive predictive value (%)	91.7	90.9	95.0	94.1	98.2
Negative predictive value (%)	81.8	90.0	75.0	81.8	52.9
Prevalence (%)	56.5	52.4	68.8	64.3	86.5

## 4. Discussion

Malignant melanoma is a tumor with an inadequate response to chemotherapy and radiation, and its incidence rate has been increasing steadily over the past 30 years.^[24,25]^ Patients with malignant melanoma that is detected at the early stage show a high survival rate. Therefore, accurate diagnostic tools for the early detection of malignant melanoma are needed. In recent years, developments in molecular analyses, genomics, and cancer biology technologies have led to the discovery of numerous new cancer biomarkers. Serum and urine biomarkers for cancer diagnosis have been of great interest because they are less invasive and the samples are easier to handle. However, effective serum and urine biomarkers for the early detection and accurate diagnosis of melanoma have not yet been identified. In this study, we found that MIA, in addition to 5-S-CD, may be an excellent biomarker for melanoma as a single factor. Additionally, we found that the combination of serum 5-S-CD, MIA, and IL-8 levels showed improved diagnostic accuracy and stratified patients with stage I/II melanoma, which was difficult using the individual factors alone.

We previously reported that serum 6-hydroxy-5-methoxyindole-2 carboxylic acid is more sensitive than 5-S-CD in diagnosing melanoma patients.^[26]^ Other reports have shown that S100B, another serum marker for melanoma, correlates better with survival than 5-S-CD.^[13,27]^ In contrast, other reports have indicated that S100B is not suitable for screening and early diagnosis because it is susceptible to blood flow and sample preservation.^[28–30]^ In this cohort, S100B levels were not significantly increased in the sera of patients with advanced melanoma (data not shown), and it was therefore excluded from the combination analysis.

In this cohort, MIA was identified as a highly sensitive biomarker. In support of our results, a previous report suggested that MIA may be sensitive enough to be used for the detection of malignant melanoma and monitoring its treatment.^[31]^ Other studies reported a significant correlation between survival and MIA levels,^[32,33]^ and serum levels of MIA were significantly reduced in stage IV melanoma patients who respond to chemotherapy,^[15]^ indicating that serum MIA levels may reflect tumor volume. Some reports showed that serum IL-8 levels increase in correlation with melanoma progression and metastatic potential of the cells.^[21–23,34]^ Our results also showed that serum IL-8 has the same diagnostic ability as 5-S-CD.

There is no doubt about the ability of 5-S-CD as a diagnostic marker, but as other reports have shown, there are false-negative patients.^[9,12,13]^ We showed that the combination of 5-S-CD, MIA, and IL-8 showed improved diagnostic capability. In addition, we used the triple marker to successfully detect patients with stage I/II melanoma (positive rate: 87.5%), which was difficult to detect with 5-S-CD alone (positive rate: 20.8%). This result suggests the importance of evaluating the disease by multiple factors with different mechanisms and indicates that the diagnostic ability of markers may be further improved by adding other factors. In addition, research has shown that combining 5-S-CD levels with other biomarkers may predict metastasis and response to treatment. For example, the combination of circulating melanoma cells and 5-S-CD detected metastasis with significantly better accuracy than the individual factors.^[35]^ Other reports showed that the lactate dehydrogenase and 5-S-CD combination may predict the response to dacarbazine treatment^[36]^ and anti-programmed death-1 antibody therapy.^[37]^ These findings indicate that it is very important to evaluate multiple factors in various combinations. This study was conducted in a small cohort of Japanese patients, and therefore it is necessary to explore the utility of the triple marker in a large cohort of different patient groups.

In summary, we have shown that the combination of 3 factors with different mechanisms, 5-S-CD, IL-8, and MIA, may enable the early diagnosis of malignant melanoma.

## Acknowledgments

We would like to thank Ms. Hideko Miyazawa for excellent technical assistance. We thank Gabrielle White Wolf, PhD, from Edanz for editing a draft of this manuscript.

## Author contributions

Conceptualization: Y.K. and H.H.

Formal analysis: Y.K. and T.H.

Funding acquisition: Y.K.

Investigation: Y.K. and H.H.

Methodology: Y.K. and S.H.

Supervision: H.H. and S.H.

Visualization: Y. K. and S.H.

Writing—original draft: Y.K., H.H., and S.H.

Writing—review & editing: Y.K., H.H., and S.H.
